# S241. COMBIMOD: A FRENCH INTEGRATIVE PROGRAMME IN PATIENTS WITH SCHIZOPHRENIA FOCUSING ON RECOVERY: PRELIMINARY RESULTS

**DOI:** 10.1093/schbul/sby018.1028

**Published:** 2018-04-01

**Authors:** Corentin Tarquis, Audrey Tanguy, sarah-Lise Farhat, Christophe Hochard, Corinne Bismuth, Corinne Gautier, Thierry Lambert, Sandrine Orens, Trang Ton, Marie-Cécile Bralet

**Affiliations:** 1 Unité CRISALID CHI Clermont de l’Oise; 2 AP-HP Paris

## Abstract

**Background:**

Cognitive remediation is a key tool in rehabilitation programs but not sufficient recovery. Meta-analyses in cognitive remediation insist on combinating programs in schizophrenia (Wykes and al.,2012; Lindenmayer and al., 2013). Futhermore, other subjective and clinical factors must be taking account in order to create a personalized rehabilitation program facilitating skills transfer in an ecological context. CRISALID (Center of cognitive rehabilitation and cognitive remediation), Clermont de l’Oise psychiatric department (Hauts de France area, France) offers an integrative care program, COMBIMod; it combines several modules of therapeutic education and of cognitive remediation, after an individual assessment, clinical, cognitive and functional (quantitative and qualitative) of each patient. A personalized and contractualized project of rehabilitation is then created with all the care partners around the patient. Objectives are: first to present COMBIMod with the description of each integrative education therapeutic programs based on cognitive deficits; then to proceed to a statistical neuropsychological comparative analysis. We postulate that using an integrative and ecological approach including remediation cognitive technics and ecological psychoeducation programs, can improve cognitive deficits.

**Methods:**

First, we described all the steps to enter COMBIMOD program since the global personalized assessments to the creation of an individualized program including cognitive remediation (Neurocognition and Social-cognition) and therapeutics education programs (MODip, MODen). We illustrated this program with patients verbatim. Then, we recruited 15 stabilized patients with schizophrenia according to DSM-IV-TR criteria. All the patients were assessed using neuropsychological tests at the baseline and after completed the program. We used Wilcoxon test analysis with level of significancy < 0,05.

**Results:**

We found significant improvement in some visuospacial memory test (p< 0,02) and executive functions (semantic verbal fluency: p<0,02; alphabetical verbal fluency: p<0,01; TMTB: p<0,003). Verbatim show some impact on the daily living and cognitive perceptions of the patient.

**Discussion:**

These preliminary results confirm the importance of an integrative rehabilitation program including personalized assessments, motivation working, mixed cognitive remediation and psychoeducation programs. In order to facilitate ecological skills transfer, rehabilitation program should focus on subjective and will assessments. Further studies must be conducted in a larger cohort to better understand recovery

